# A genetic variant study of bortezomib-induced peripheral neuropathy in Chinese multiple myeloma patients

**DOI:** 10.32604/or.2023.043922

**Published:** 2024-04-23

**Authors:** YAN ZHANG, HEYANG ZHANG, JING WANG, XIN WEI, YI QU, FENG XU, LIJUN ZHANG

**Affiliations:** Department of Hematology, The First Affiliated Hospital of China Medical University, Shenyang, 110001, China

**Keywords:** Multiple Myeloma, Peripheral neuropathy, Bortezomib, Bortezomib-induced peripheral neuropathy, Next-generation sequencing, *MTHFR*, Serum Hcy

## Abstract

**Background:**

Bortezomib results in peripheral neuropathy (PN) in approximately 50% of patients, during multiple myeloma (MM) treatment, a complication known as Bortezomib-induced peripheral neuropathy (BIPN). The drug response varies among individuals. Genetic factor may play an important role in BIPN.

**Methods:**

A next-generation sequencing (NGS) panel containing 1659 targets from 233 genes was used to identify risk variants for developing BIPN in 204 MM patients who received bortezomib therapy. mRNA expression of *MTHFR* and *ALDH1A1* in 62 peripheral blood samples was detected by real-time quantitative PCR (RT-qPCR). Serum homocysteine (Hcy) levels were detected in 40 samples by chemiluminescent microparticle immunoassay (CMIA).

**Results:**

Compared with the non-BIPN group (n = 89), a total of 8 significantly associated single nucleotide polymorphisms (SNPs) were identified in the BIPN group (n = 115): *MTHFR* (rs1801131, rs1801133, rs17421511), *EPHX1* (rs1051740), *MME* (rs2016848), *ALDH1A1* (rs6151031), *HTR7* (rs1935349) and *CYP2A6* (rs8192720). The mRNA expression level of *MTHFR* in newly diagnosed patients with peripheral neuritis after treatment (NP group) was lower than that of newly diagnosed patients without peripheral neuritis after treatment (NnP group) (1.70 ± 0.77 *vs*. 2.81 ± 0.97, ***p*** = 0.009). Serum Hcy levels were significantly higher in BIPN group than in non-BIPN group (11.66 ± 1.79 μmol/L *vs*. 8.52 ± 3.29 μmol/L, ***p*** = 0.016) and healthy controls (11.66 ± 1.79 μmol/L *vs*. 8.55 ± 2.13 μmol/L, ***p*** ≤ 0.001).

**Conclusion:**

*CYP2A6, EPHX1, MTHFR, ALDH1A1, HTR7, MME* and BIPN are linked in Chinese MM patients. BIPN is more likely to occur in patients with lower *MTHFR* mRNA expression, which might result in higher serum Hcy levels.

## Introduction

Multiple myeloma (MM) is a hematological malignancy caused by malignant proliferation of plasma cells, and currently ranks second in hematological disease incidence [1]. Bortezomib is a proteasome inhibitor, that mainly inhibits Nuclear factor κB (NF-κB) signaling [2], downregulates growth factor receptors, suppresses adhesion molecule expression and inhibits angiogenesis [3]. It is the first proteasome inhibitor approved for the market. In 2003, bortezomib was approved by the US Food and Drug Administration (FDA) for treatment of MM. It can effectively inhibit proliferation of myeloma cells and promote apoptosis of myeloma cells, offering new hope for treatment of MM. At present, bortezomib (B) and lenalidomide (R) combined with dexamethasone (D) are widely used in China, and this regimen is currently the first choice of standard therapy for MM patients [4].

Although, patients with MM benefit greatly with use of bortezomib with respect to symptoms, patients receiving bortezomib have some common adverse effects, including peripheral neuropathy, gastrointestinal symptoms, thrombocytopenia, and postural hypotension. Among the adverse effects, bortezomib-induced peripheral neuropathy (BIPN) is the most serious, with a high incidence of approximately 50% [5–7], and has attracted the attention of clinicians. BIPN mainly develops as limb numbness or pain, typically manifesting as glove and sock type sensory disorders [8]. Patients with severe peripheral neuropathy need to have a reduced dose of bortezomib or stop its use, which might have adverse effects on prognosis and quality of life. Despite the established dose and clinical risk factors for BIPN, interindividual variation and susceptibility have been observed to be associated with the development of drug-related adverse events under similar treatment plans. It has been reported that this unpredictability is partly attributable to genetic differences [9] Single nucleotide polymorphisms (SNPs) and gene expression profiles may play an important role in BIPN. Studies on genetic variant correlations with BIPN have been reported in Western countries. Several studies have identified genes with variant SNPs involved in different functions, such as bortezomib metabolism (*CYP1A1, CYP1A2, CYP1B1, CYP2C19*, and *CYP2S1*) [10–13], protein transport (*ABCC1* and *ABCC6*) [14], nervous system development, structure and function (*TCF4, ABI3BP BASP1, NRN1, TMEM107, POGZ, EDN1, ANKRD45, PRKG1*, and *KCNA5*), proteasome function and proteolytic process (*BTRC PSMB1, PSMB4, F2*) [11,14,15], regulation of RNA transcription (*MALAT1, EGR1, FMR1, PKNOX1*), protein, steroid, and lipid synthesis (*CYP17A1, HK2 TDO2*, and *CBS*) [16,17] and inflammatory and immune responses (*CTLA4, HIST1H2BC, CX3CR1, ERAP2, CFH, MX2, FCRL3, FCRL2, C1orf106NEB, IL17RD, IL10RA*, and *NFATC4*) [11,14,15]. However, there have been no similar genetic studies in Chinese MM patients.

Moreover, there are no suitable clinical predictors or biomarkers to identify patients at high risk of developing BIPN. We propose that genotyping MM patients for the SNPs examined in this study will help in identifying which genotypes are susceptible to BIPN and explore the possible mechanism of BIPN.

## Materials and Methods

### Patients

From May 2019 to December 2021, 204 Chinese MM patients who received bortezomib therapy at the Department of Hematology in the First Affiliated Hospital of China Medical University were recruited. This study was approved by the Ethics Committee of the First Affiliated Hospital of China Medical University, the Ethical Committee process number is [2023] 523. Written inform consent was obtained from all participants involved in the study.

Inclusion criteria: (1) Chinese patients from northeast China, and of Han ethnicity; (2) diagnosed with MM without other hematological diseases or peripheral nerve-related diseases before treatment; and (3) receiving treatment with bortezomib (standard dose of 1.3 mg/m^2^) ≥3 cycles or less than 3 cycles of treatment because of BIPN intolerance.

The patients received induction therapy with bortezomib-dexamethasone (BD), bortezomib-thalidomide-dexamethasone (BTD) or bortezomib-lenalidomide -dexamethasone (BRD). No other chemotherapy drugs were used during follow-up. The grade of peripheral neuritis after bortezomib treatment was determined according to the National Cancer Institute Common Terminology Criteria for Adverse Events version 5.0 [18].

### DNA sequencing and genotyping

Genomic DNA was isolated from peripheral blood nucleated cells using QIAamp DNA Blood Kit (Qiagen, Hilden,Germany) according to the manufacturer’s instructions. Quantification of the DNA concentration was performed using Qubit® dsDNA HS Assay Kit (Yeasen, Shanghai, China) according the manufacturer’s protocol. The NGS panel, which contains 1659 targets from 233 drug metabolism-related genes, including drug-metabolizing enzymes, drug transporters, gene expression regulators, and additional genes of potential pharmacological interest (shown in Suppl. Table S1), was amplified with 1659 pairs of custom-designed primers. Genotyping was performed using MultipSeq® Custom Panel Kit (IGMU228XV1) (iGeneTech, Beijing, China). Protocols were carried out according to the manufacturer’s recommendations. High-quality sequencing reads were specifically captured using the amplicon sequencing method (E-Seq Mecidal, Beijing, China) and sequenced using an Illumina HiSeq X ten (Illumina, Inc., CA, USA) at high depth with 150 bp paired-end reads. Burrows Wheeler Aligner (BWA-Version: 0.7.5a-r405) was used for alignment (GRCh38; hg38). Genome Analysis Toolkit (GATK, version 4.1) was used for indel realignment, quality score recalibration, variant calling, and genotyping (using Haplotype Caller). The operating system is linux.

### RNA isolation and RT-qPCR

Total RNA was isolated from the peripheral blood of MM patients using TRIzol reagent (Invitrogen), according to the manufacturer’s instructions. Total RNA was reverse transcribed to cDNA using Prime-Script RT Master Mix (Invitrogen). For detection of *MTHFR* and *ALDH1A1* levels in the peripheral bloodof patients and controls, RT-qPCR was conducted using SYBR Green Master Mix (TaKaRa) and an ABI 7500 Real-Time PCR system (Applied Biosystems). The following conditions were used: one cycle of 95°C for 30 s, followed by 40 cycles of a two-step cycling program (95°C for 5 s, 60°C for 34 s). mRNA expression of target genes was calculated by the 2^−ΔΔCt^ method and normalized to *GAPDH* mRNA expression. The primers for the *MTHFR* gene were as follows: forward:5′-TCACCATCAACTCACAGCCC-3′; reverse:5′-AGAAGTGCTTCCGCTGTCTC-3′. The primers for the *ALTH1A1* gene were as follows: forward:5′-AGTGCCCCTTTGGTGGATTC-3′; reverse:5′-AAGAGCTTCTCTCCACTCTTG-3′. The primers for the *GAPDH* gene were as follows: forward:5′-GTCTCCTCTGACTTCAACAGCG-30; reverse: 50-ACCACCCTGTTGCTGTAGCCAA-3′.

### Detection of serum homocysteine (Hcy)

Serum Hcy levels were detected by chemiluminescent microparticle immunoassay (CMIA). The reagents were purchased from Abbott Germany Biotechnology Co., Ltd. (Germany), and the operations were carried out in strict accordance with the Homocysteine Reagent kit instructions.

### Statistical analysis

Quantitative differences between groups were compared using *t*-test or Mann-Whitney u-test (*Hb, LDH, Cr, ß2-MG*, Ca) depends on whether the date conforms to a normal distribution, and chi-square (*χ2*) test was used for count date with SPSS 22.0 software. Sequencing data were sorted and analyzed by R version 3.4, and the required text processing was performed using Perl language. The machine learning method of R software was used to calculate the variation frequency (the number of variant bases (or the number of variant genotypes)/the total number of bases (the total number of genotypes)). Minor allele frequency (MAF) <0.05 was not considered to meet the research criteria and not included. Variant analysis between the BIPN group and the non-BIPN group was performed using R statistical software version 3.4 to estimate odds ratios (ORs) and *p* values. Results with a *p* value < 0.05 were considered statistically significant and selected for subsequent analysis. Variants with OR > 1 and *p* ≤ 0.01 from the above results were selected as SNPs most related to BIPN. A binary logistic model was used for multivariate analysis with SPSS 22.0 software, and *p* < 0.05 was considered statistically significant.

## Results

### Clinical characteristics

A total of 204 MM patients who met the inclusion criteria and for whom sequencing was successful were enrolled in this study, including 115 patients with BIPN and 89 patients without BIPN. There was no significant difference in clinical information, such as sex and age, between the BIPN group and the non-BIPN group (*p* > 0.05). The information is shown in Table 1.

**Table 1:** Clinical characteristics of the study population

Characteristics	BIPN (n = 115)	Non-BIPN (n = 89)	*p*
Sex male/female	68/47	46/43	0.321
Age^a)^	60.02 ± 9.21	61.76 ± 8.45	0.168
Diabetes	17	9	0.399
Smoke	38	20	0.118
Intemperance	36	25	0.647
Wbc^b)^	6.07 ± 3.82	5.80 ± 2.76	0.615
Hb^c)^	95	87	0.584
Plt^d)^	181.01 ± 81.38	185.78 ± 96.27	0.732
LDH^e)^	166.5	168	0.855
Cr^f)^	77	86	0.802
ß2-MG^g)^	5.39	5.21	0.563
Ca^h)^	2.28	2.27	0.939
Immune globulin			
IgA^i)^	25	19	0.995
IgD^j)^	12	6	0.552
IgG^k)^	59	40	0.434
Nonsecretion	17	22	0.48
Light-chain immunoglobulin			
K	56	40	0.673
λ	50	41	0.571
Nonsecretion	9	7	0.991

Abbreviations: a): age, (mean, years); b): white blood cell, (mean, ×109/L); c): hemoglobin, (median, g/L); d): platelet, (mean, ×109/L); e): lactic dehydrogenase, (median, U/L); f): creatinine, (median, μmol/l); g): beta2 microglobulin, (median, mg/l); h): calcium, (median, mmol/l) i): immunoglobulin A; j): immunoglobulin D; k): immunoglobulin G.

### Genotyping results

Sequencing analysis was performed for 204 MM samples (115 BIPN and 89 non-BIPN). Eighty-six differential records with a *p* value < 0.05 were found, corresponding to 44 SNPs. The information is shown in Suppl. Table S2. OR values of the above 86 records were calculated, and 37 records with both *p* value < 0.05 and OR > 1 were obtained, corresponding to 13 genes. The information is shown in Suppl. Table S3.

The most significantly different SNPs between the BIPN group and non-BIPN group were detected by the cutoff value of *p* ≤ 0.01 and OR > 1, among the 37 records. Finally, 16 records most related to BIPN were obtained, involving 8 SNPs and 6 genes. The variation information and genotype variation frequency of the two groups are shown in Table 2. The results were as follows: rs1801131 in *MTHFR* (T > G, AF: 0.217 (BIPN) *vs*. 0.096 (non-BIPN), OR = 2.631, *p* = 0.001; T/T > T/G, AF: 0.383 *vs*. 0.191, OR = 2.743, *p* = 0.002); rs1801133 in *MTHFR* (A > G, AF: 0.678 *vs*. 0.466, OR = 2.413, *p* ≤ 0.001; A/A > G/G, AF: 0.530 *vs*. 0.393, OR = 5.364, *p* = 0.001); rs17421511 in *MTHFR* (G > A, AF: 0.152 *vs*. 0.039, OR = 4.385, *p* ≤ 0.001; G/G > G/A, AF: 0.304 *vs*. 0.079, OR = 5.132, *p* ≤ 0.001); rs1051740 in *EPHX1* (C > T, AF: 0.700 *vs*. 0.472, OR = 2.611, *p* ≤ 0.001;T/C > T/T, AF: 0.496 *vs*. 0.191, OR = 3.566, *p* ≤ 0.001); rs2016848 in *MME* (G > A, AF: 0.348 *vs*. 0.124, OR = 3.782, *p* = 0.001; G/G > A/G, AF: 0.330 *vs*. 0.112, OR = 3.960, *p* ≤ 0.001); rs6151031 in *ALDH1A1* (GCTGGTGAGGAGAGAACC > G, AF: 0.061 *vs*. 0.011, OR = 5.704, *p* = 0.010; GCTGGTGAGGAGAGAACC/GCTGGTGAGGAGAGAACC > G/GCTGGTGAGGAGAGAACC, AF: 0.122 *vs*. 0.022, OR = 6.027, *p* = 0.009); rs1935349 in *HTR7* (C > T, AF: 0.343 *vs*. 0.208, OR = 1.994, *p* = 0.003; C/C > T/T, AF: 0.139 *vs*. 0.022, OR = 8.313, *p* = 0.002); rs8192720 in *CYP2A6* (G/A > A/A, AF: 0.130 *vs*. 0, OR infinity, *p* = 0.001; G/G > A/A, AF: 0.130 *vs*. 0, OR infinity, *p* ≤ 0.001) (associated variant sites are shown in Suppl. Figs. S1 (A–H).

**Table 2:** List of the observed Variations and Frequencies most significant between BIPN and non-BIPN group

SNP^a)^	Ref^b)^	Alt^c)^	MAF^d)^(BIPN)	MAF(non-BIPN)	OR^e)^	*p* value	SNP type	Gene
rs1801131	T	G	0.217	0.096	2.631	0.001	Exonic	*MTHFR*
	T/T	T/G	0.383	0.191	2.743	0.002	Exonic	*MTHFR*
rs1801133	A	G	0.678	0.466	2.413	≤0.001	Exonic	*MTHFR*
	A/A	G/G	0.530	0.393	5.364	0.001	Exonic	*MTHFR*
rs17421511	G	A	0.152	0.039	4.385	≤0.001	Intronic	*MTHFR*
	G/G	G/A	0.304	0.079	5.132	≤0.001	Intronic	*MTHFR*
rs1051740	C	T	0.700	0.472	2.611	≤0.001	Exonic	*EPHX1*
	T/C	T/T	0.496	0.191	3.566	≤0.001	Exonic	*EPHX1*
rs2016848	G	A	0.348	0.124	3.782	0.001	Intronic	*MME*
	G/G	A/G	0.330	0.112	3.960	≤0.001	Intronic	*MME*
rs6151031	GCT*^f)^	G	0.061	0.011	5.704	0.010	Upstream	*ALDH1A1*
	GCT*/GCT*	G/GCT*	0.122	0.022	6.027	0.009	Upstream	*ALDH1A1*
rs1935349	C	T	0.343	0.208	1.994	0.003	Intronic	*HTR7*
	C/C	T/T	0.139	0.022	8.313	0.002	Intronic	*HTR7*
rs8192720	G/A	A/A	0.130	0	Inf^g)^	0.001	Exonic	*CYP2A6*
	G/G	A/A	0.130	0	inf	≤0.001	Exonic	*CYP2A6*

Abbreviations: a): single nucleotide polymorphisms; b): reference sequence; c): alternative; d): minor allele frequencies; e): odds ratios; f): GCTGGTGAGGAGAGAACC; g): infinity.

To evaluate the association between genetic polymorphisms and BIPN, we compared the genotype distribution of the BIPN group and non-BIPN group. The genotype analysis between the two groups were shown in Table 3. The variant genotypes in the BIPN group (rs1801131, T/G, n = 44 (38.3%); rs1801133, G/G, n = 61 (53.0%); rs1801133, G/A, n = 34 (29.6%); rs17421511, G/A, n = 35 (30.4%); rs1051740, T/T, n = 57 (49.6%); rs2016848, A/G, n = 38 (33.0%); rs6151031, GCTGGTGAGGAGAGAACC/G, n = 14 (12.2%); rs1935349, T/T, n = 16 (13.9%); rs8192720, AA, n = 15 (13.1%)) were higher than those in the non-BIPN group, and all of these genotypes might be risk genotypes for BIPN.

**Table 3:** Frequencies of genetic polymorphisms in BIPN and non-BIPN groups

Genotype	BIPN^a)^	non-BIPN	OR^b)^ (95% CI)	*p* value
	(n = 115)	(n = 89)		
*MTHFR*				
rs1801131				
TT	68 (59.1%)	72 (80.9%)	1	
TG	44 (38.3%)	17 (19.1%)	2.74 (1.43–5.25)	0.002
GG	3 (2.6%)	0 (0%)	Inf	0.12
TG+GG	47 (40.9%)	17 (19.1)	2.93 (1.53–5.59)	0.001
rs1801133				
AA	20 (17.4%)	41 (46.1%)	1	
GG	61 (53.0%)	35 (39.3%)	3.57 (1.82–7.03)	≤0.001
GA	34 (29.6%)	13 (14.6%)	5.36 (2.33–12.34)	0.001
GG+GA	95 (82.6%)	48 (53.9%)	4.06 (2.15–7.67)	≤0.001
rs17421511				
GG	80 (69.6%)	82 (92.1%)	1	
GA	35 (30.4%)	7 (7.9%)	5.13 (2.15–12.21)	≤0.001
AA	0 (0%)	0 (0%)	–	–
GA+AA	35 (30.4%)	7 (7.9%)	5.13 (2.15–12.21)	≤0.001
*EPHX1*				
rs1051740				
TC	47 (40.9%)	50 (56.2%)	1	
CC	11 (9.6%)	22 (24.7%)	0.53 (0.23–1.22)	0.158
TT	57 (49.6%)	17 (19.1%)	3.57 (1.82–6.99)	≤0.001
CC+TT	68 (59.1%)	39 (43.8%)	1.86 (1.06–3.25)	0.034
*MME*				
rs2016848				
GG	75 (65.2%)	78 (87.6%)	1	0.618
AA	2 (1.8%)	1 (1.1%)	2.08 (0.18–23.42)	
AG	38 (33.0%)	10 (11.2%)	3.96 (1.84–8.50)	≤0.001
AG+AA	40 (34.8%)	11 (12.3)	3.78 (1.81–7.92)	≤0.001
*ALDH1A1*				
rs6151031				
GCT*^c)^/GCT*	101 (87.8%)	87 (97.8%)	1	
GCT*/G	14 (12.2%)	2 (2.2%)	6.03 (1.33–27.27)	0.009
G/G	0 (0%)	0 (0%)	–	–
GCT*/G+G/G	14 (12.2%)	2 (2.2%)	6.03 (1.33–27.27)	0.009
*HTR7*				
rs1935349				
CC	52 (45.2%)	54 (60.7%)	1	
CT	47 (40.9%)	33 (37.1%)	1.48 (0.82–2.66)	0.235
TT	16 (13.9%)	2 (2.2%)	8.31 (1.82–37.93)	0.002
CT+TT	63 (54.8%)	35 (39.3%)	1.87(1.07–3.28)	0.034
*CYP2A6*				
rs8192720				
GG	69 (60.0%)	61 (68.5%)	1	
AG	31 (26.9%)	28 (31.5%)	0.98 (0.53–1.81)	1
AA	15 (13.1%)	0 (0%)	Inf^d)^	≤0.001
AG+AA	46 (40.0%)	28 (31.5%)	1.45 (0.81–2.60)	0.241

Abbreviations: a): Bortezomib-induced Peripheral Neuropathy; b): odds ratios; c): GCTGGTGAGGAGAGAACC; d): infinity.

**Figure 1:**
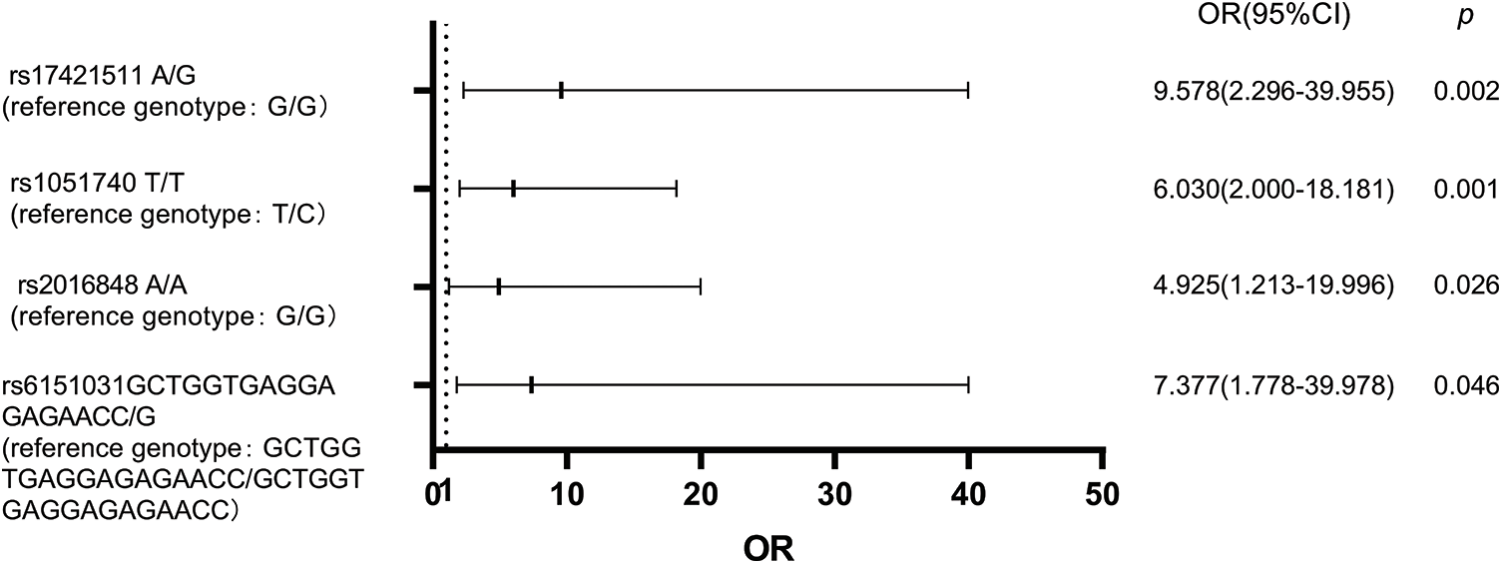
Logistic regression model forest diagram showing the risk of BIPN according to combined genotypes of genetic variants.

A multivariate logistic regression model was used to analyze the above eight SNPs of each genotype. It was found that the risk of BIPN for the G/A genotype at rs17421511 was 9.578 times that of the G/G genotype (*p* = 0.002, OR = 9.578 (95% CI 2.296–39.955)), the T/T genotype at rs1051740 was 6.030 times that of the T/C genotype (*p* = 0.001,OR = 6.030 (95% CI 2.000–18.181)), A/A genotype at rs2016848 was 4.925 times that of the G/G genotype (*p* = 0.026, OR = 4.925 (95% CI 1.213–19.996)), and the GCTGGTGAGGAGAGAACC/G genotype at rs6151031 was 7.377 times that of the GCTGGTGAGGAGAGAACC/GCTGGTGAGGAGAGAACC genotype (*p* = 0.046, OR = 7.377 (95% CI 1.778–39.978)). A logistic regression model forest diagram is shown in Fig. 1.

### MTHFR and ALDH1A1 mRNA expression

mRNA expression of *MTHFR* and *ALDH1A1* in 62 peripheral blood samples was detected by RT-PCR. Among the 6 genes with BIPN-related SNPs detected by panel sequencing, these two genes were expressed in peripheral blood, whereas the other 4 genes had expression levels that were low and not detectable. Among the 62 samples, there were 26 cases among the newly diagnosed untreated MM patients (N group), 19 cases among the retreated patients who were treated after bortezomib (R group) and 17 cases among the healthy persons as the control group (C group). Among the 26 patients in the N group, 8 developed peripheral neuritis after bortezomib treatment (NP group); the other 18 did not develop peripheral neuritis after bortezomib treatment (NnP group). Of the 19 patients in the R group, 12 developed peripheral neuritis after bortezomib treatment (RP group), and the other 7 did not (RnP group).

**Figure 2:**
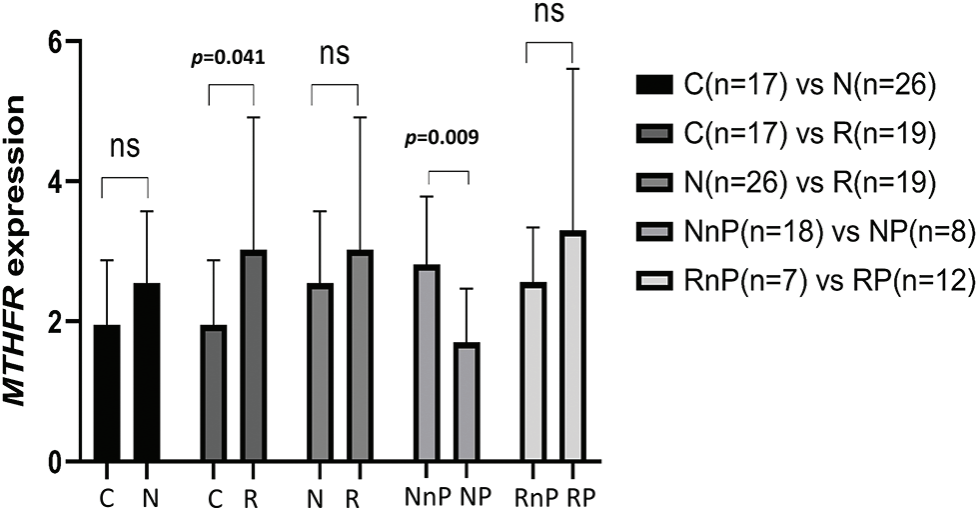
Comparison of *MTHFR* expression in different group. N group: newly diagnosed untreated MM patients; R group: retreated patients who treated after bortezomib; C group: the healthy persons as control group; NP group: N group developed peripheral neuritis after bortezomib treatment; NnP group: N group did not develop peripheral neuritis after bortezomib treatment; RP group: R group developed peripheral neuritis after bortezomib treatment; RnP group: R group without peripheral neuritis after bortezomib treatment.

(1) There was no significant difference in the expression level of *MTHFR* mRNA in the N group compared with the C group (2.55 ± 1.02 *vs*. 1.95 ± 0.92, *p* = 0.050). The mRNA expression level of *MTHFR* in the R group was higher than that in the C group (3.02 ± 1.89 *vs*. 1.95 ± 0.92, *p* = 0.041), and the difference was statistically significant. The mRNA expression level of *MTHFR* in the N group was similar to that in the R group (2.55 ± 1.0 *vs*. 3.02 ± 1.89, *p* = 0.264). The mRNA expression level of *MTHFR* in the NP group was lower than that in the NnP group (1.70 ± 0.77 *vs*. 2.81 ± 0.97, *p* = 0.009), and the difference was statistically significant. There was no difference in the mRNA expression level of *MTHFR* between the RP group and RnP group (3.30 ± 2.31 *vs*. 2.56 ± 0.78, *p* = 0.426). The results are shown in Fig. 2.

(2) The mRNA expression level of *ALDH1A1* in the N group was not different from that in the C group (6.74 ± 4.76 *vs*. 4.63 ± 5.67, *p* = 0.182); the mRNA expression level of *ALDH1A1* in the R group was higher than that in the C group (11.15 ± 11.92 *vs*. 4.63 ± 5.67, *p* = 0.048), and the difference was statistically significant. However, there was no difference in the mRNA expression level of *ALDH1A1* between the N group and R group (6.74 ± 4.76 *vs*. 11.15 ± 11.92, *p* = 0.080). The mRNA expression level of *ALDH1A1* in the NP group was not different from that in the NnP group (5.15 ± 2.26 *vs*. 6.92 ± 5.27, *p* = 0.375); the mRNA expression level of *ALDH1A1* in the RP and RnP groups did not differ (8.15 ± 6.67 *vs*. 16.24 ± 17.23, *p* = 0.159).

### Serum Hcy level

**Figure 3:**
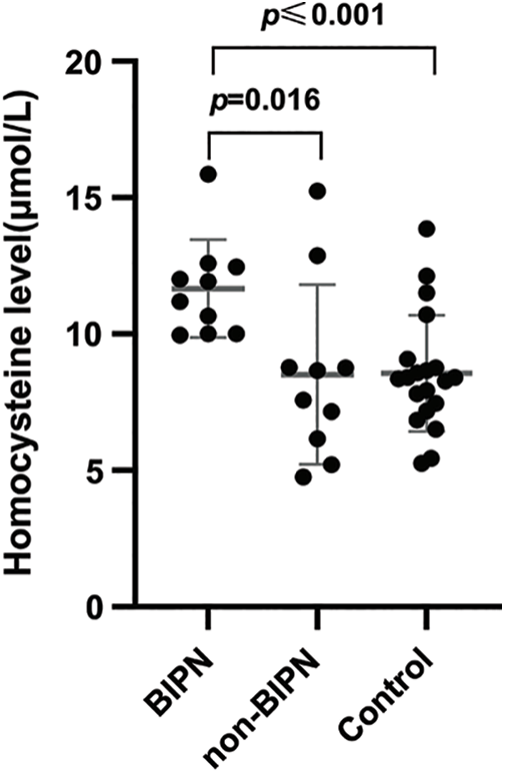
Comparison of HCY levels among different groups. The serum Hcy levels in the BIPN group were dramatically higher than non-BIPN group and control group.

Forty samples were assessed for serum Hcy, including from 10 BIPN patients, 10 non-BIPN patients and 20 healthy subjects. All subjects had normal renal function and no cardiovascular or cerebrovascular disease. Serum Hcy levels of the BIPN group were higher than those of the non-BIPN group, and the difference was statistically significant (11.66 ± 1.79 μmol/L *vs*. 8.52 ± 3.29 μmol/L, *p* = 0.016). Compared with healthy subjects, serum Hcy levels of BIPN patients were higher (11.66 ± 1.79 μmol/L *vs*. 8.55 ± 2.13 μmol/L, *p* ≤ 0.001), but there was no statistically significant difference between the non-BIPN group and healthy subjects (8.52 ± 3.29 μmol/L *vs*. 8.55 ± 2.13 μmol/L, *p* = 0.969). The results are shown in Fig. 3.

## Discussion

Peripheral neurotoxicity is the most common nonhematologic toxicity in clinical chemotherapy and can caused by proteasome inhibitors such as bortezomib [19]. BIPN is considered to be one of the most serious and unpredictable long-term side effects of chemotherapy for multiple myeloma, with adverse effects on patient quality of life [20,21]. Some patients develop peripheral neuropathy (PN) while others do not, despite the same dose of bortezomib and the same regimen according to the body surface area; hence, individual differences in BIPN development exist. At present, such individual differences are believed to be related to genetic factors and unpredictability [9]. Statistically, 15% to 30% of the variation in drug response is attributed to differences in genotype or SNP [22]. How to identify patients who may develop severe side effects caused by individual genetic differences, which are not related to drug dosage, is a clinical difficulty and challenge.

Genetic studies have been reported in other countries. A study including 139 blood samples from MM patients treated with bortezomib identified rs4553808, which is located in the *CTLA4* gene, as being associated with BIPN [15]. Broyl et al. [23] used a panel composed of 3404 SNPs to analyze 186 MM patients treated with bortezomib induction therapy and found that *RHOBTB2* an enzyme-encoding gene involved in drug-induced apoptosis, the *CPT1C* gene involved in mitochondrial function, and the *SOX8* gene involved in peripheral nervous system development are associated with early-onset BIPN. The authors suggested that specific gene expression profiles may be associated with risk of BIPN as well as its severity. To identify susceptibility sites related to BIPN, Magrangeas et al. [17] conducted a genome-wide study of 583 MM patients and found that specific sites of *PKNOX1* and *CBS* related to nerve pain are associated with peripheral neuritis. Azoulay et al. [24] conducted genetic studies on peripheral neuritis in 45 patients with multiple myeloma treated with bortezemib and concluded that SNPs of the *BDNF* gene are associated with peripheral neuritis caused by chemotherapy. Campo et al. [25] identified bortezomib-induced peripheral neuritis in association with 4q34.3 (rs6552496), 5q14.1 (rs12521798), 16q23.3 (rs8060632) and 18q21.2 (rs17748074) in 646 MM patients, and all of these loci are involved in nervous system development and signal transduction. García-Sanz et al. [10] analyzed exon single nucleotide polymorphisms in 172 MM patients treated with bortezomib and thalidomide and observed development of chemotherapy complications in patients under similar treatment conditions, and the results showed that *PLCG2* rs45443101 may be associated with adverse drug reactions. These studies have demonstrated the genetic basis of neurotoxicity and identified SNPs in genes or adjacent genes that affect the development and function of the nervous system.

In this study, three SNPs located in the *MTHFR* gene (rs17421511, rs1801131, rs1801133) were found to be associated with BIPN. Gene polymorphism can affect or inhibit the function of downstream transcription factors, genes or proteins in a variety of ways [26]. The change in MTHFR activity can cause disturbance of methionine synthesis, reducing the level of S-adenosine methionine, which can cause nerve myelin damage and change the level of serum Hcy [27–29]. Increased serum Hcy level has a direct cytotoxic effect on the nervous system, which leads to neuropathy [30]. This study found that the T/G genotype of rs1801131, G/G genotype of rs1801133 and G/A genotype of rs17421511 may be risk genotypes for BIPN. We also found that BIPN was more likely to occur in patients with lower levels of *MTHFR* mRNA before bortezomib treatment. Furthermore, levels of serum Hcy were higher in BIPN than non-BIPN patients. Our previous experiments found that patients with abnormal renal function had higher serum Hcy levels and Kumar et al. [31] summarized that patients with cardiovascular or cerebrovascular diseases or end-stage renal disease might have abnormal Hcy levels. To reduce study bias, we enrolled MM patients with normal renal function and no cardiovascular or cerebrovascular disease for Hcy level detection. BIPN patients might show increased serum Hcy levels through abnormal *MTHFR* function in the above mechanisms compared with non-BIPN, which might be one of the factors leading to BIPN.

Gene variation might affect the treatment of bortezomib in tumors such as MM through different kinds of mechanisms. Treatment with bortezomib induces the expression of proteins related with the endoplasmic reticulum secretory pathway, the activation of caspase and Ca^2+^ homeostasis,which leads to cell death [32]. The aforementioned variation of *MTHFR* can affect MTHFR activity to affect the process or speed of methionine conversion metabolism, which can increase the level of Hcy and also promote Ca^2+^ homeostasis disorder caused by bortezomib [27–29,33]. The variation of *ALDH1A1* affects its regulatory function in the RA-RARα signaling pathway [34], which might weaken the repairment of injured nerves after bortezomib treatment. CYP2A6 catalyzes many reactions in drug metabolism and lipid metabolism [35]. The variation of *EPHX1* may participate in the oxidative stress response caused by mitochondrial damage after bortezomib treatment [36]. The variations of *HTR7* and *MME* have not been reported on the mechanism related to bortezomib, but have been verified to be associated with neurological diseases [37,38].

As clinical information and examinations cannot determine whether patients will develop BIPN, it is important to find new methods to predict BIPN. Such as genetic factors. PN is a disease with complex mechanisms, and multiple genes are involved. BIPN is related to the accumulated dose of bortezomib, the duration of treatment, and combined drugs such as thalidomide [20]. In our study, the patients enrolled were all of Han ethnicity in Northeast China, which is the majority ethnic group in China, and none had neuropathy before, as patients with neuropathy were excluded. These patients were treated with a unified dose of bortezomib (1.3 mg/m^2^) in accordance with Chinese guidelines for myeloma treatment [4] to minimize the bias caused by different doses. Some patients with thalidomide treatment might also experience the occurrence or aggravation of PN, which might affect the results of this study. However, for the different time points and cycles between bortezomib and thalidomide, we selected patients with PN who were more inclined to be caused by bortezomib to minimize bias. Samples for which it could not be distinguished whether PN was caused by bortezomib or thalidomide were excluded from the study. However, it cannot be denied that use of thalidomide can aggravate the grade of BIPN, which is a shortcoming of this study. The SNPs that were discovered in this study have not been reported in other studies related to BIPN. The reason may be that the gene and SNP compositions of panel sequencing used by scholars in other countries are different from ours. In addition, differences in treatment plan and drug dosage in different countries might have led to differences in the results of the studies. More importantly, there are ethnic genetic differences between the Chinese populations in Asia and populations in Europe and America.

To our knowledge, this is the first study to describe the association between BIPN and gene polymorphisms in Chinese MM patients in an Asian context, and our study reveals a possible mechanism related to BIPN and *MTHFR*. But our research also has some limitations. We did not do further SNP functional analysis; not all BIPN associated genes were expressed in peripheral blood, but in liver or other tissues, so we could not obtain samples for detection; the sample size of *MTHFR* mRNA, *ALDH1A1* mRNA and serum Hcy are relatively small, and we will expand the sample size and do functional analysis for further study in the future. It may provide an experimental basis for research in this field, and offer a reference for evaluating pharmacogenomic data based on Asians for further research.

## Conclusion

Our study reported 8 genetic variants most related to BIPN in Chinese MM patients. And BIPN is more likely to occur in patients with lower *MTHFR* mRNA expression, which might result in higher serum Hcy levels. It may provide a screening measure to predict BIPN in MM patients before treatment.

## Supplementary Materials

Supplementary Figure 1 The most significantly different SNPs associated variant sites between the BIPN group and non-BIPN group. A: variation in rs1801131 (T>G) of MTHFR; B: variation in rs1801133(A>G) of *MTHFR*; C: variation in rs17421511(G>A) of *MTHFR*; D: variation in rs1051740(C>T) of *EPHX1*. E: variation in rs2016848 (G>A) of *MME*; F: variation in rs6151031(GCTGGTGAGGAGAGAACC>G) of *ALDH1A1*; G: variation in rs1935349(C>T) of *HTR7*; H: variation in rs8192720 (G>A) of *CYP2A6*.

## Data Availability

The datasets used and/or analyzed during the current study are available from the corresponding author on reasonable request.

## Supplementary Materials

The supplementary material is available online at https://doi.org/10.32604/or.2023.043922.

## References

[1] van de Donk, N., Pawlyn, C., Yong, K. L. (2021). Multiple myeloma. Lancet*,* 397 *(*10272*),* 410–427. 10.1016/S0140-6736(21)00135-5; 33516340

[2] Cai, K., Na, W., Guo, M., Xu, R., Wang, X. et al. (2019). Targeting the cross-talk between the hedgehog and NF-κB signaling pathways in multiple myeloma. Leukemia & Lymphoma*,* 60 *(*3*),* 772–781. 10.1080/10428194.2018.1493727; 30644322

[3] Gandolfi, S., Laubach, J. P., Hideshima, T., Chauhan, D., Anderson, K. C. et al. (2017). The proteasome and proteasome inhibitors in multiple myeloma. Cancer Metastasis Reviews*,* 36 *(*4*),* 561–584. 10.1007/s10555-017-9707-8; 29196868

[4] Chinese Hematology Association, Chinese Society of Hematology, Chinese Myeloma Committee-Chinese Hematology Association (2020). The guidelines for the diagnosis and management of multiple myeloma in China (2020 revision). Chinese Journal of Internal Medicine*,* 59 *(*5*),* 341–346 (In Chinese). 10.3760/cma.j.cn112138-20200304-00179; 32370461

[5] Bechakra, M., Nieuwenhoff, M. D., van Rosmalen, J., Groeneveld, G. J., Scheltens-de Boer, M. et al. (2018). Clinical, electrophysiological, and cutaneous innervation changes in patients with bortezomib-induced peripheral neuropathy reveal insight into mechanisms of neuropathic pain. Molecular Pain*,* 14*,* 1744806918797042. 10.1177/1744806918797042; 30152246 PMC6113731

[6] Velasco, R., Petit, J., Clapés, V., Verdú, E., Navarro, X. et al. (2010). Neurological monitoring reduces the incidence of bortezomib-induced peripheral neuropathy in multiple myeloma patients. Journal of the Peripheral Nervous System*,* 15 *(*1*),* 17–25. 10.1111/j.1529-8027.2010.00248.x; 20433602

[7] Lakshman, A., Modi, M., Prakash, G., Malhotra, P., Khadwal, A. et al. (2017). Evaluation of bortezomib-induced neuropathy using total neuropathy score (reduced and clinical versions) and NCI CTCAE v4.0 in newly diagnosed patients with multiple myeloma receiving Bortezomib-based induction. Clinical Lymphoma, Myeloma & Leukemia*,* 17 *(*8*),* 513–519.E1. 10.1016/j.clml.2017.06.035; 28842138

[8] Kerckhove, N., Collin, A., Condé, S., Chaleteix, C., Pezet, D. et al. (2018). Neuropathies périphériques chimio-induites: Symptomatologie et épidémiologie. Bulletin du Cancer*,* 105 *(*11*),* 1020–1032. 10.1016/j.bulcan.2018.07.009 30244980

[9] Chang, W. C., Tanoshima, R., Ross, C., Carleton, B. C. (2021). Challenges and opportunities in implementing pharmacogenetic testing in clinical settings. Annual Review of Pharmacology and Toxicology*,* 61*,* 65–84. 10.1146/annurev-pharmtox-030920-025745; 33006916

[10] García-Sanz, R., Corchete, L. A., Alcoceba, M., Chillon, M. C., Jiménez, C. et al. (2017). Prediction of peripheral neuropathy in multiple myeloma patients receiving bortezomib and thalidomide: A genetic study based on a single nucleotide polymorphism array. Hematological Oncology*,* 35 *(*4*),* 746–751. 10.1002/hon.2337; 27605156

[11] Velasco, R., Alberti, P., Bruna, J., Psimaras, D., Argyriou, A. A. (2019). Bortezomib and other proteosome inhibitors-induced peripheral neurotoxicity: From pathogenesis to treatment. Journal of the Peripheral Nervous System*,* 24 *(*S2*),* S52–S62. 10.1111/jns.12338; 31647153

[12] Zhou, W., An, G., Jian, Y., Guo, H., Chen, W. (2015). Effect of CYP2C19 and CYP3A4 gene polymorphisms on the efficacy of bortezomib-based regimens in patients with multiple myeloma. Oncology Letters*,* 10 *(*2*),* 1171–1175. 10.3892/ol.2015.3294; 26622646 PMC4509025

[13] Vangsted, A. J., Søeby, K., Klausen, T. W., Abildgaard, N., Andersen, N. F. et al. (2010). No influence of the polymorphisms CYP2C19 and CYP2D6 on the efficacy of cyclophosphamide, thalidomide, and bortezomib in patients with Multiple Myeloma. BMC Cancer*,* 10*,* 404. 10.1186/1471-2407-10-404; 20684753 PMC2922196

[14] Campo, C., Da Silva Filho, M. I., Weinhold, N., Goldschmidt, H., Hemminki, K. et al. (2017). Genetic susceptibility to bortezomib-induced peripheral neuroropathy: Replication of the reported candidate susceptibility loci. Neurochemical Research*,* 42 *(*3*),* 925–931. 10.1007/s11064-016-2007-9; 27422265

[15] Favis, R., Sun, Y., van de Velde, H., Broderick, E., Levey, L. et al. (2011). Genetic variation associated with bortezomib-induced peripheral neuropathy. Pharmacogenetics and Genomics*,* 21 *(*3*),* 121–129. 10.1097/FPC.0b013e3283436b45; 21228734

[16] Corthals, S. L., Kuiper, R., Johnson, D. C., Sonneveld, P., Hajek, R. et al. (2011). Genetic factors underlying the risk of bortezomib induced peripheral neuropathy in multiple myeloma patients. Haematologica*,* 96 *(*11*),* 1728–1732. 10.3324/haematol.2011.041434; 21791469 PMC3208695

[17] Magrangeas, F., Kuiper, R., Avet-Loiseau, H., Gouraud, W., Guérin-Charbonnel, C. et al. (2016). A Genome-wide association study identifies a novel locus for bortezomib-induced peripheral neuropathy in european patients with multiple myeloma. Clinical Cancer Research*,* 22 *(*17*),* 4350–4355. 10.1158/1078-0432.CCR-15-3163; 27060151 PMC5010494

[18] Common Terminology Criteria for Adverse Events (CTCAE) (2017). CTCAE_v5_Quick_Reference_5x7. pdf. https://ctep.cancer.gov/protocoldevelopment/electronic_applications/ctc.htm (accessed on 01/05/2019)

[19] Sioka, C., Kyritsis, A. P. (2009). Central and peripheral nervous system toxicity of common chemotherapeutic agents. Cancer Chemotherapy and Pharmacology*,* 63 *(*5*),* 761–767. 10.1007/s00280-008-0876-6; 19034447

[20] Argyriou, A. A., Iconomou, G., Kalofonos, H. P. (2008). Bortezomib-induced peripheral neuropathy in multiple myeloma: A comprehensive review of the literature. Blood*,* 112 *(*5*),* 1593–1599. 10.1182/blood-2008-04-149385; 18574024

[21] Argyriou, A. A., Zolota, V., Kyriakopoulou, O., Kalofonos, H. P. (2010). Toxic peripheral neuropathy associated with commonly used chemotherapeutic agents. Journal of BUON*,* 15 *(*3*),* 435–446; 20941808

[22] Eichelbaum, M., Ingelman-Sundberg, M., Evans, W. E. (2006). Pharmacogenomics and individualized drug therapy. Annual Review of Medicine*,* 57*,* 119–137. 10.1146/annurev.med.56.082103.104724; 16409140

[23] Broyl, A., Corthals, S. L., Jongen, J. L., van der Holt, B., Kuiper, R. et al. (2010). Mechanisms of peripheral neuropathy associated with bortezomib and vincristine in patients with newly diagnosed multiple myeloma: A prospective analysis of data from the HOVON-65/GMMG-HD4 trial. The Lancet Oncology*,* 11 *(*11*),* 1057–1065. 10.1016/S1470-2045(10)70206-0; 20864405

[24] Azoulay, D., Giryes, S., Nasser, R., Sharon, R., Horowitz, N. A. (2019). Prediction of chemotherapy-induced peripheral neuropathy in patients with lymphoma and myeloma: The roles of brain-derived neurotropic factor protein levels and a gene polymorphism. Journal of Clinical Neurology*,* 15 *(*4*),* 511–516. 10.3988/jcn.2019.15.4.511; 31591840 PMC6785478

[25] Campo, C., da Silva Filho, M. I., Weinhold, N., Mahmoudpour, S. H., Goldschmidt, H. et al. (2018). Bortezomib-induced peripheral neuropathy: A genome-wide association study on multiple myeloma patients. Hematological Oncology*,* 36 *(*1*),* 232–237. 10.1002/hon.2391; 28317148

[26] Pang, G. S., Wang, J., Wang, Z., Lee, C. G. (2009). Predicting potentially functional SNPs in drug-response genes. Pharmacogenomics*,* 10 *(*4*),* 639–653. 10.2217/pgs.09.12; 19374519

[27] Khalighi, K., Cheng, G., Mirabbasi, S., Khalighi, B., Wu, Y. et al. (2018). Opposite impact of methylene tetrahydrofolate reductase C677T and Methylene tetrahydrofolate reductase A1298C gene polymorphisms on systemic inflammation. Journal of Clinical Laboratory Analysis*,* 32 *(*5*),* e22401. 10.1002/jcla.22401; 29396861 PMC6817214

[28] Zeng, Q., Li, F., Xiang, T., Wang, W., Ma, C. et al. (2017). Influence of food groups on plasma total homocysteine for specific MTHFR C677T genotypes in Chinese population. Molecular Nutrition & Food Research*,* 61 *(*2*),* 1600351. 10.1002/mnfr.201600351; 27515258 PMC5297973

[29] Renard, D., Dutray, A., Remy, A., Castelnovo, G., Labauge, P. (2009). Subacute combined degeneration of the spinal cord caused by nitrous oxide anaesthesia. Neurological Sciences*,* 30 *(*1*),* 75–76. 10.1007/s10072-009-0013-2; 19169627

[30] Ala, O. A., Akintunde, A. A., Ikem, R. T., Kolawole, B. A., Ala, O. O. et al. (2017). Association between insulin resistance and total plasma homocysteine levels in type 2 diabetes mellitus patients in south west Nigeria. Diabetes & Metabolic Syndrome*,* 11*,* S803–S809. 10.1016/j.dsx.2017.06.002; 28610915

[31] Kumar, A., Palfrey, H. A., Pathak, R., Kadowitz, P. J., Gettys, T. W. et al. (2017). The metabolism and significance of homocysteine in nutrition and health. Nutrition & Metabolism*,* 14*,* 78. 10.1186/s12986-017-0233-z; 29299040 PMC5741875

[32] Landowski, T. H., Megli, C. J., Nullmeyer, K. D., Lynch, R. M., Dorr, R. T. (2005). Mitochondrial-mediated disregulation of Ca^2+^ is a critical determinant of Velcade (PS-341/bortezomib) cytotoxicity in myeloma cell lines. Cancer Research*,* 65 *(*9*),* 3828–3836. 10.1158/0008-5472.CAN-04-3684; 15867381

[33] Ji, X. W., Lyu, H. J., Zhou, G. H., Wu, B., Zhu, Y. Y. et al. (2021). Physcion, a tetra-substituted 9,10-anthraquinone, prevents homocysteine-induced endothelial dysfunction by activating Ca^2+^- and Akt-eNOS-NO signaling pathways. Phytomedicine*,* 81*,* 153410. 10.1016/j.phymed.2020.153410; 33285470

[34] Kumar, S., Duester, G. (2011). SnapShot: Retinoic acid signaling. Cell*,* 147 *(*6*),* 1422–1422.e1. 10.1016/j.cell.2011.11.034; 22153083 PMC3242729

[35] Hoffman, S. M., Nelson, D. R., Keeney, D. S. (2001). Organization, structure and evolution of the CYP2 gene cluster on human chromosome 19. Pharmacogenetics*,* 11 *(*8*),* 687–698. 10.1097/00008571-200111000-00007; 11692077

[36] Gautheron, J., Jéru, I. (2020). The multifaceted role of epoxide hydrolases in human health and disease. International Journal of Molecular Sciences*,* 22 *(*1*),* 13. 10.3390/ijms22010013; 33374956 PMC7792612

[37] Crispino, M., Volpicelli, F., Perrone-Capano, C. (2020). Role of the serotonin receptor 7 in brain plasticity: From development to disease. International Journal of Molecular Sciences*,* 21 *(*2*),* 505. 10.3390/ijms21020505; 31941109 PMC7013427

[38] Hong, D., Fang, P., Yao, S., Chen, J., Zhang, X. et al. (2019). Variants in MME are associated with autosomal-recessive distal hereditary motor neuropathy. Annals of Clinical and Translational Neurology*,* 6 *(*9*),* 1728–1738. 10.1002/acn3.50868; 31429185 PMC6764622

