# Supplementary material for: A genetic variant study of bortezomib-induced peripheral neuropathy in Chinese multiple myeloma patients
Source: Oncol Res. 2024 Apr 23;32(5):955–63. doi: 10.32604/or.2023.043922 (PMC11055991; doi:10.32604/or.2023.043922)
Supplement: Supplementary file 3 [file OncolRes-32-43922-s001.docx]

**Table S1 The list of 233 drug metabolism-related genes in the NGS panel**

| ABCA1 | ABCB1 | ABCC2 | ABCC3 | ABCC4 | ABCC8 | ABCG2 | ABCG8 | ACE | ACE2 |
| --- | --- | --- | --- | --- | --- | --- | --- | --- | --- |
| ADAMTS1 | ADD1 | ADH1B | ADIPOQ | ADORA1 | ADRA1A | ADRB1 | ADRB2 | ADRB3 | AGT |
| AGTR1 | ALDH2 | ALDH3A1 | AOX1 | APOA5 | APOB | APOC3 | APOE | ATIC | ATM |
| ATP1A1 | ATP5E | B4GALT2 | BCMO1 | CALU | CAPN10 | CBR1 | CBR3 | CCND1 | CD3EAP |
| CEP68 | CES1 | CETP | CFTR | CHRNA3 | CLCN2 | CLCN6 | CLCNKA | CLCNKB | CLGN |
| COMT | CRP | CTH | CTLA4 | CTNNB1 | CYBA | CYP11B1 | CYP17A1 | CYP1A2 | CYP1B1 |
| CYP21A2 | CYP27B1 | CYP2A6 | CYP2B6 | CYP2C19 | CYP2C8 | CYP2C9 | CYP2D6 | CYP2J2 | CYP3A4 |
| CYP3A5 | CYP3A7 | CYP4A11 | CYP4F11 | CYP4F2 | DNMT3A | DPYD | DPYS | EDN1 | EDNRB |
| EFS | EGFR | EPHX1 | ERCC1 | ERCC2 | ESR1 | F13A1 | F5 | FAAH | FABP1 |
| FCGR2A | FCGR3A | FGF23 | FGFR4 | FLT3 | FMO3 | FOLH1 | FPGS | FTO | FUT2 |
| G6PC2 | G6PD | GABRA1 | GC | GGCX | GGH | GLP1R | GNAS | GNB3 | GRK4 |
| GRK5 | GSTM3 | HAS3 | HMGCR | HSD11B1 | HSD11B2 | IMPDH1 | IMPDH2 | IRS1 | ITGA2 |
| ITGB3 | ITPA | KCNA5 | KCNH2 | KCNJ11 | KCNJ5 | KCNMB1 | KDR | KIF6 | KLK1 |
| KRAS | LASS5 | LDLR | LEPR | LIG3 | LPL | LSS | MCM8 | MED12L | MS4A3 |
| MSH3 | MTHFD1 | MTHFR | MTR | MTRR | MUTYH | MYLIP | NAT2 | NEUROD1 | NOD2 |
| NOS3 | NPHS1 | NPPA | NQO1 | NQO2 | NR1H3 | NR1I2 | NR3C1 | NR3C2 | NUDT15 |
| OPRK1 | ORM1 | P2RY1 | P2RY12 | PAX4 | PEAR1 | PLCG1 | PNPLA3 | PON1 | PPARG |
| PRCP | PRKCH | PTGS2 | RARG | RENBP | SCAP | SCARB1 | SCN2A | SCNN1A | SCNN1B |
| SCNN1G | SDHB | SELE | SERPINA6 | SHMT1 | SLC12A1 | SLC12A3 | SLC14A2 | SLC16A7 | SLC19A1 |
| SLC22A1 | SLC22A16 | SLC22A2 | SLC22A3 | SLC23A2 | SLC24A3 | SLC28A3 | SLC2A2 | SLC30A8 | SLC30A9 |
| SLC47A1 | SLC6A12 | SLCO1B1 | SLCO1B3 | SLCO2A1 | SOD2 | STRN | SULT1A3 | SULT1A4 | SUMO4 |
| TAPBP | TCN2 | TF | TLR3 | TLR4 | TNFRSF11A | TP53 | TPMT | TSC1 | UGT1A1 |
| UGT1A3 | UGT1A4 | UGT1A6 | UGT1A8 | UGT1A9 | UGT2B15 | UGT2B7 | UMPS | VDR | VKORC1 |
| WNK4 | XRCC1 | ZBTB22 |  |  |  |  |  |  |  |
