# Supplementary material for: A genetic variant study of bortezomib-induced peripheral neuropathy in Chinese multiple myeloma patients
Source: Oncol Res. 2024 Apr 23;32(5):955–63. doi: 10.32604/or.2023.043922 (PMC11055991; doi:10.32604/or.2023.043922)
Supplement: Supplementary file 4 [file OncolRes-32-43922-s002.docx]

**Table S2 SNP records of 86 cases** **satisfying the conditions of *p* value<0.05**

| \| SNP \| Gene \| Ref \| Alt \| *p* value \| MAF(BIPN) \| MAF(nonBIPN) \| SNP  Type \| \| --- \| --- \| --- \| --- \| --- \| --- \| --- \| --- \| |
| --- | --- | --- | --- | --- | --- | --- | --- | --- |
| \| rs3114017 \| *ABCG2* \| C/T \| C/C \| 0.027 \| 0.588 \| 0.333 \| intronic \| \| --- \| --- \| --- \| --- \| --- \| --- \| --- \| --- \| \| rs1967308 \| *ADCY9* \| C/C \| T/C \| 0.030 \| 0.426 \| 0.205 \| intronic \| \| rs11091046 \| *AGTR2* \| C/C \| A/A \| 0.035 \| 0.404 \| 0.182 \| UTR3 \| \| rs11091046 \| *AGTR2* \| C \| A \| 0.007 \| 0.422 \| 0.238 \| UTR3 \| \| rs2163204 \| *APOB* \| T/T \| T/G \| 0.028 \| 0.169 \| 0.025 \| exonic \| \| rs11288208 \| *CDKN2B-AS1* \| AT/A \| AT/AT \| 0.029 \| 0.536 \| 0.286 \| ncRNA_intronic \| \| N \| *COL26A1* \| A \| ATT \| 0.034 \| 0.265 \| 0.040 \| intronic \| \| N \| *COL26A1* \| AT/A \| AT/ATT \| 0.034 \| 0.265 \| 0.040 \| intronic \| \| rs10105643 \| *CYP11B1* \| C \| A \| 0.013 \| 0.189 \| 0.064 \| intronic \| \| rs10105643 \| *GML* \| C \| A \| 0.013 \| 0.189 \| 0.064 \| intronic \| \| rs10105643 \| *CYP11B1* \| C/C \| A/C \| 0.033 \| 0.322 \| 0.128 \| intronic \| \| rs10105643 \| *GML* \| C/C \| A/C \| 0.033 \| 0.322 \| 0.128 \| intronic \| \| rs8192720 \| *AC008537.1* \| G/A \| A/A \| 0.001 \| 0.130 \| 0.000 \| exonic \| \| rs8192720 \| *CYP2A6* \| G/A \| A/A \| 0.001 \| 0.130 \| 0.000 \| exonic \| \| rs8192720 \| *AC008537.1* \| G/G \| A/A \| 0.000 \| 0.188 \| 0.000 \| exonic \| \| rs8192720 \| *CYP2A6* \| G/G \| A/A \| 0.000 \| 0.130 \| 0.000 \| exonic \| \| rs3213422 \| *DHODH* \| C/C \| A/C \| 0.021 \| 0.516 \| 0.263 \| exonic \| \| rs1051740 \| *EPHX1* \| T/C \| T/T \| 0.000 \| 0.496 \| 0.191 \| exonic \| \| rs1131873 \| *EPHX1* \| G/G \| G/A \| 0.040 \| 0.583 \| 0.359 \| exonic \| \| rs1051740 \| *EPHX1* \| C \| T \| 0.000 \| 0.700 \| 0.472 \| exonic \| \| rs846910 \| *HSD11B1* \| G/G \| A/A \| 0.040 \| 0.250 \| 0.040 \| ncRNA_intronic \| \| rs846910 \| *HSD11B1-AS1* \| G/G \| A/A \| 0.040 \| 0.250 \| 0.040 \| ncRNA_intronic \| \| rs846908 \| *HSD11B1-AS1* \| G/G \| A/A \| 0.034 \| 0.325 \| 0.083 \| ncRNA_intronic \| \| rs3753519 \| *HSD11B1* \| C/C \| T/T \| 0.038 \| 0.457 \| 0.158 \| ncRNA_intronic \| \| rs3753519 \| *HSD11B1-AS1* \| C/C \| T/T \| 0.038 \| 0.457 \| 0.158 \| ncRNA_intronic \| \| rs701950 \| *HSD11B1-AS1* \| A/A \| G/A \| 0.028 \| 0.500 \| 0.257 \| ncRNA_intronic \| \| rs701950 \| *HSD11B1-AS1* \| A \| G \| 0.007 \| 0.339 \| 0.153 \| ncRNA_intronic \| \| rs3753519 \| *HSD11B1* \| C \| T \| 0.042 \| 0.473 \| 0.309 \| ncRNA_intronic \| \| rs3753519 \| *HSD11B1-AS1* \| C \| T \| 0.042 \| 0.473 \| 0.309 \| ncRNA_intronic \| \| rs1935349 \| *HTR7* \| C/C \| T/T \| 0.002 \| 0.139 \| 0.022 \| intronic \| \| rs1935349 \| *HTR7* \| C \| T \| 0.003 \| 0.343 \| 0.208 \| intronic \| \| rs2835904 \| *KCNJ6* \| T/C \| C/C \| 0.003 \| 0.405 \| 0.074 \| intronic \| \| rs2835904 \| *KCNJ6* \| T/T \| C/C \| 0.033 \| 0.436 \| 0.125 \| intronic \| \| rs860795 \| *AP001407.1* \| C/G \| G/G \| 0.044 \| 0.350 \| 0.111 \| intronic \| \| rs860795 \| *KCNJ6* \| C/G \| G/G \| 0.044 \| 0.350 \| 0.111 \| intronic \| \| rs2237895 \| *KCNQ1* \| A/A \| C/C \| 0.020 \| 0.263 \| 0.037 \| intronic \| \| rs2237895 \| *KCNQ1* \| A \| C \| 0.013 \| 0.362 \| 0.195 \| intronic \| \| rs2016848 \| *MME* \| G/G \| A/G \| 0.000 \| 0.330 \| 0.112 \| intronic \| \| rs2016846 \| *MME* \| T/T \| G/T \| 0.013 \| 0.308 \| 0.122 \| intronic \| \| rs2016848 \| *MME* \| G \| A \| 0.001 \| 0.348 \| 0.124 \| intronic \| \| rs2016846 \| *MME* \| T \| G \| 0.021 \| 0.636 \| 0.061 \| intronic \| \| rs1801131 \| *MTHFR* \| T \| G \| 0.001 \| 0.217 \| 0.096 \| exonic \| \| rs17421511 \| *MTHFR* \| G/G \| G/A \| 0.000 \| 0.304 \| 0.079 \| intronic \| \| rs1801131 \| *MTHFR* \| T/T \| T/G \| 0.002 \| 0.383 \| 0.191 \| exonic \| \| rs1801133 \| *MTHFR* \| A/A \| G/G \| 0.001 \| 0.530 \| 0.393 \| exonic \| \| rs7533315 \| *MTHFR* \| C/C \| T/C \| 0.005 \| 0.361 \| 0.098 \| intronic \| \| rs17421511 \| *MTHFR* \| G \| A \| 0.000 \| 0.152 \| 0.039 \| intronic \| \| rs7533315 \| *MTHFR* \| C \| T \| 0.009 \| 0.273 \| 0.049 \| intronic \| \| rs12121543 \| *MTHFR* \| C \| A \| 0.011 \| 0.206 \| 0.075 \| intronic \| \| rs12121543 \| *MTHFR* \| C/C \| C/A \| 0.024 \| 0.361 \| 0.150 \| intronic \| \| rs1801133 \| *MTHFR* \| A \| G \| 0.000 \| 0.678 \| 0.466 \| exonic \| \| rs2279239 \| *NR1H3* \| C/C \| T/T \| 0.038 \| 0.324 \| 0.053 \| intronic \| \| rs61824877 \| *LINC00862* \| G/G \| G/A \| 0.033 \| 0.448 \| 0.231 \| intergenic \| \| rs61824877 \| *LINC00862* \| G \| A \| 0.015 \| 0.308 \| 0.159 \| intergenic \| \| rs594141 \| *PAX4* \| G/G \| A/A \| 0.008 \| 0.344 \| 0.042 \| intronic \| \| rs594141 \| *PAX4* \| G \| A \| 0.005 \| 0.423 \| 0.232 \| intronic \| \| rs1993945 \| *AC022414.1* \| T \| A \| 0.002 \| 0.221 \| 0.044 \| intronic \| \| rs1993945 \| *PDE8B* \| T \| A \| 0.002 \| 0.221 \| 0.044 \| intronic \| \| rs1993945 \| *AC022414.1* \| T/T \| A/T \| 0.034 \| 0.261 \| 0.061 \| intronic \| \| rs1993945 \| *PDE8B* \| T/T \| A/T \| 0.034 \| 0.261 \| 0.061 \| intronic \| \| rs4704397 \| *AC022414.1* \| A \| G \| 0.007 \| 0.154 \| 0.037 \| intronic \| \| rs4704397 \| *PDE8B* \| A \| G \| 0.007 \| 0.154 \| 0.037 \| intronic \| \| rs4704397 \| *AC022414.1* \| A/A \| G/A \| 0.021 \| 0.254 \| 0.073 \| intronic \| \| rs4704397 \| *PDE8B* \| A/A \| G/A \| 0.021 \| 0.254 \| 0.073 \| intronic \| \| rs56106044 \| *PEAR1* \| A/G \| G/G \| 0.012 \| 0.389 \| 0.084 \| intronic \| \| rs56106044 \| *PEAR1* \| A/A \| G/G \| 0.030 \| 0.483 \| 0.091 \| intronic \| \| rs1799808 \| *PROC* \| C/T \| T/T \| 0.023 \| 0.444 \| 0.200 \| upstream \| \| rs1254598 \| *PTGER2* \| G/A \| A/A \| 0.028 \| 0.344 \| 0.083 \| UTR5 \| \| rs2887284 \| *REN* \| C/C \| C/A \| 0.012 \| 0.482 \| 0.211 \| intronic \| \| rs2368564 \| *REN* \| C/C \| C/T \| 0.019 \| 0.475 \| 0.231 \| intronic \| \| rs2887284 \| *REN* \| C \| A \| 0.018 \| 0.303 \| 0.150 \| intronic \| \| rs2368564 \| *REN* \| C \| T \| 0.015 \| 0.308 \| 0.159 \| intronic \| \| rs10887990 \| *SDHB* \| T/T \| C/C \| 0.011 \| 0.417 \| 0.091 \| intronic \| \| rs10887990 \| *SDHB* \| T/C \| C/C \| 0.036 \| 0.366 \| 0.100 \| intronic \| \| rs10887990 \| *SDHB* \| T \| C \| 0.012 \| 0.452 \| 0.275 \| intronic \| \| rs77846911 \| *SLC12A1* \| T/T \| T/C \| 0.018 \| 0.351 \| 0.125 \| intronic \| \| rs78681617 \| *SLC12A1* \| G/G \| G/C \| 0.018 \| 0.351 \| 0.128 \| intronic \| \| rs8032941 \| *SLC12A1* \| T/T \| T/C \| 0.015 \| 0.377 \| 0.150 \| intronic \| \| rs77846911 \| *SLC12A1* \| T \| C \| 0.008 \| 0.230 \| 0.085 \| intronic \| \| rs78681617 \| *SLC12A1* \| G \| C \| 0.013 \| 0.230 \| 0.088 \| intronic \| \| rs8032941 \| *SLC12A1* \| T \| C \| 0.011 \| 0.238 \| 0.098 \| intronic \| \| rs1484548 \| *SLC12A1* \| G/G \| T/G \| 0.037 \| 0.579 \| 0.351 \| intronic \| \| rs11221518 \| *TP53AIP1* \| G/G \| G/A \| 0.040 \| 0.349 \| 0.154 \| UTR3 \| \| rs7299460 \| *VDR* \| T/T \| C/C \| 0.040 \| 0.543 \| 0.269 \| intronic \| \| rs7299460 \| *VDR* \| T \| C \| 0.019 \| 0.525 \| 0.346 \| intronic \| \| rs6151031 \| *ALDH1A1* \| GCTGGTGAGGAGAGAACC \| G \| 0.010 \| 0.061 \| 0.011 \| unstream  transcript \| \| rs6151031 \| *ALDH1A1* \| GCTGGTGAGGAGAGAACC / GCTGGTGAGGAGAGAACC \| GCTGGTGAGGAGAGAACC /G \| 0.009 \| 0.122 \| 0.022 \| unstream  transcript \| |

Abbreviations: SNP: single nucleotide polymorphisms; Ref: reference sequence; Alt: alternative; MAF: minor allele frequencies; N: no named.
