# Supplementary material for: A genetic variant study of bortezomib-induced peripheral neuropathy in Chinese multiple myeloma patients
Source: Oncol Res. 2024 Apr 23;32(5):955–63. doi: 10.32604/or.2023.043922 (PMC11055991; doi:10.32604/or.2023.043922)
Supplement: Supplementary file 5 [file OncolRes-32-43922-s003.docx]

**Table S3 Records of 37 SNPs in 13 genes satisfying the conditions of *p* value<0.05 and OR>1**

| \| SNP \| Gene \| Ref \| Alt \| OR \| *p* value \| MAF(BIPN) \| MAF(non-BIPN) \| SNP  Type \| \| --- \| --- \| --- \| --- \| --- \| --- \| --- \| --- \| --- \| |
| --- | --- | --- | --- | --- | --- | --- | --- | --- | --- |
| \| rs2163204 \| *APOB* \| T/T \| T/G \| 7.833 \| 0.028 \| 0.169 \| 0.025 \| exonic \| \| --- \| --- \| --- \| --- \| --- \| --- \| --- \| --- \| --- \| \| rs8192720 \| *CYP2A6* \| G/A \| A/A \| Inf \| 0.001 \| 0.130 \| 0.000 \| exonic \| \| rs8192720 \| *CYP2A6* \| G/G \| A/A \| Inf \| 0.000 \| 0.130 \| 0.000 \| exonic \| \| rs3213422 \| *DHODH* \| C/C \| A/C \| 2.954 \| 0.021 \| 0.516 \| 0.263 \| exonic \| \| rs1051740 \| *EPHX1* \| T/C \| T/T \| 3.566 \| 0.000 \| 0.496 \| 0.191 \| exonic \| \| rs1131873 \| *EPHX1* \| G/G \| G/A \| 2.476 \| 0.040 \| 0.583 \| 0.524 \| exonic \| \| rs1051740 \| *EPHX1* \| C \| T \| 2.611 \| 0.000 \| 0.700 \| 0.472 \| exonic \| \| rs1935349 \| *HTR7* \| C/C \| T/T \| 8.313 \| 0.002 \| 0.139 \| 0.022 \| intronic \| \| rs1935349 \| *HTR7* \| C \| T \| 1.994 \| 0.003 \| 0.343 \| 0.208 \| intronic \| \| rs2237895 \| *KCNQ1* \| A/A \| C/C \| 9.039 \| 0.020 \| 0.263 \| 0.037 \| intronic \| \| rs2237895 \| *KCNQ1* \| A \| C \| 2.327 \| 0.013 \| 0.362 \| 0.195 \| intronic \| \| rs2016848 \| *MME* \| G/G \| A/G \| 3.960 \| 0.000 \| 0.330 \| 0.112 \| intronic \| \| rs2016846 \| *MME* \| T/T \| G/T \| 3.642 \| 0.013 \| 0.338 \| 0.061 \| intronic \| \| rs2016848 \| *MME* \| G \| A \| 3.782 \| 0.001 \| 0.348 \| 0.124 \| intronic \| \| rs2016846 \| *MME* \| T \| G \| 3.122 \| 0.021 \| 0.169 \| 0.061 \| intronic \| \| rs1801131 \| *MTHFR* \| T \| G \| 2.631 \| 0.001 \| 0.217 \| 0.096 \| exonic \| \| rs17421511 \| *MTHFR* \| G/G \| G/A \| 5.132 \| 0.000 \| 0.304 \| 0.079 \| intronic \| \| rs1801131 \| *MTHFR* \| T/T \| T/G \| 2.743 \| 0.002 \| 0.383 \| 0.191 \| exonic \| \| rs1801133 \| *MTHFR* \| A/A \| G/G \| 5.364 \| 0.001 \| 0.530 \| 0.393 \| exonic \| \| rs7533315 \| *MTHFR* \| C/C \| T/C \| 4.780 \| 0.005 \| 0.344 \| 0.240 \| intronic \| \| rs17421511 \| *MTHFR* \| G \| A \| 4.385 \| 0.000 \| 0.152 \| 0.039 \| intronic \| \| rs7533315 \| *MTHFR* \| C \| T \| 4.024 \| 0.009 \| 0.172 \| 0.543 \| intronic \| \| rs12121543 \| *MTHFR* \| C \| A \| 3.191 \| 0.011 \| 0.206 \| 0.073 \| intronic \| \| rs12121543 \| *MTHFR* \| C/C \| C/A \| 3.162 \| 0.024 \| 0.361 \| 0.037 \| intronic \| \| rs1801133 \| *MTHFR* \| A \| G \| 2.413 \| 0.000 \| 0.678 \| 0.466 \| exonic \| \| rs56106044 \| *PEAR1* \| A/G \| G/G \| 14.730 \| 0.012 \| 0.389 \| 0.084 \| intronic \| \| rs56106044 \| *PEAR1* \| A/A \| G/G \| 8.900 \| 0.030 \| 0.483 \| 0.091 \| intronic \| \| rs1799808 \| *PROC* \| C/T \| T/T \| 3.159 \| 0.023 \| 0.444 \| 0.200 \| upstream \| \| rs2887284 \| *REN* \| C/C \| C/A \| 3.445 \| 0.012 \| 0.482 \| 0.211 \| intronic \| \| rs2368564 \| *REN* \| C/C \| C/T \| 2.977 \| 0.019 \| 0.475 \| 0.150 \| intronic \| \| rs2887284 \| *REN* \| C \| A \| 2.456 \| 0.018 \| 0.303 \| 0.231 \| intronic \| \| rs2368564 \| *REN* \| C \| T \| 2.350 \| 0.015 \| 0.308 \| 0.159 \| intronic \| \| rs10887990 \| *SDHB* \| T/T \| C/C \| 6.928 \| 0.011 \| 0.417 \| 0.091 \| intronic \| \| rs10887990 \| *SDHB* \| T/C \| C/C \| 5.073 \| 0.036 \| 0.366 \| 0.100 \| intronic \| \| rs10887990 \| *SDHB* \| T \| C \| 2.163 \| 0.012 \| 0.452 \| 0.275 \| intronic \| \| rs6151031 \| *ALDH1A1* \| GCTGGTGAGGAGAGAACC \| G \| 5.704 \| 0.010 \| 0.061 \| 0.011 \| unstream  transcript \| \| rs6151031 \| *ALDH1A1* \| GCTGGTGAGGAGAGAACC / GCTGGTGAGGAGAGAACC \| GCTGGTGAGGAGAGAACC /G \| 6.027 \| 0.009 \| 0.122 \| 0.022 \| unstream  transcript \| |

Abbreviations: SNP: single nucleotide polymorphisms; Ref: reference sequence; Alt: alternative; MAF: minor allele frequencies；OR: odds ratios.
